# Rhabdoid meningioma with a history of Budd-Chiari syndrome: a case report and review of the literature

**DOI:** 10.3389/fonc.2023.1209244

**Published:** 2023-07-11

**Authors:** Ying Zeng, Jing Zhang, Wei Jian, Yong Zhang, Ying Yang, Rongqing Li, Qiaofen Fu

**Affiliations:** ^1^ Department of Radiation Oncology, First Affiliated Hospital of Kunming Medical University, Kunming, China; ^2^ Department of Pathology, First Affiliated Hospital of Kunming Medical University, Kunming, China

**Keywords:** rhabdoid meningioma, Budd-Chiari syndrome, meningioma, central nervous system tumour, liver cirrhosis

## Abstract

**Background:**

Rhabdoid meningioma and Budd-Chiari syndrome are both extremely rare, and there is no report describing the two diseases occurring in the same patient thus far. Herein, we showed an unusual case of rhabdoid meningioma with a history of Budd-Chiari syndrome.

**Case presentation:**

The man was found to have abnormal liver function during physical examination in 2016 at 36 and was not paid attention to it. In 2019, he went to Beijing YouAn Hospital Affiliated to Capital Medical University for the decompensation of cirrhosis and was diagnosed with Budd-Chiari syndrome, subsequent angiography of the inferior vena cava combined with balloon dilatation were performed, the anticoagulation and hepatoprotective therapy were performed for a long time. When he turned 40 who had magnetic resonance imaging (MRI) that showed a left frontotemporal lobe space-occupying lesion, and postoperative pathological examination confirmed rhabdoid meningioma. He underwent surgery and postoperative adjuvant radiotherapy, but then he developed severe psychiatric symptoms and eventually succumbed to a lung infection two months after treatment.

**Conclusions:**

Budd-Chiari syndrome and Rhabdoid meningiomas are both extremely rare diseases. To the best of our knowledge, there is no report that the two rare diseases occurred in the same patient, and this is the first case. However, whether there is any link between the two diseases is unclear, more researches are needed to confirm it in the future.

## Introduction

Meningiomas are the most common subtype of central nervous system (CNS) tumours. Most meningiomas show World Health Organization (WHO) benign grade 1 histopathologic features, while WHO grade 2 and 3 meningiomas are less frequently observed (1, 2). Thus, meningiomas are associated with a usually benign clinical outcome. Rhabdoid meningiomas (RMs) are a rare tumour subtype of meningiomas accounting for 1% to 3% of all intracranial meningiomas (1, 3, 4), and they usually present with unique histopathologic characteristics and were first reported in 1998 (5, 6). In the WHO 2021 classification of CNS tumours, RM is defined based on the presence of rhabdoid cells and is classified as a WHO grade 3 tumour (1). As an unusual variant of meningioma, RM is highly invasive and has a high recurrence rate, poor prognosis, and poor survival (3, 4, 6).

Budd-Chiari syndrome (BCS) is defined as an obstruction of the hepatic venous outflow track in the absence of cardiac or pericardial diseases, also known as hepatic venous outflow obstruction (HVOTO) (7). It is a rare and potentially fatal heterogeneous disease with a prevalence of 1 case per million inhabitants a year (8), and which characterised by hepatic venous outflow obstruction (9, 10). BCS can be divided into primary and secondary according to the aetiology. Primary BCS is often associated with a variety of thrombophilia, including primary myeloproliferative neoplasms, paroxysmal nocturnal haemoglobinuria, hereditary thrombophilia, antiphospholipid antibodies, and high-risk conditions (11). Among them, myeloproliferative neoplasm (MPN) is the most common aetiology (12). Secondary BCS is mainly related to tumour invasion and compression. Long-term anticoagulation is indicated regardless of the underlying disease (7). The survival rates in treated patients range from 42 to 100% depending on the aetiology and the presence of risk factors, including the Child−Pugh score, sodium and creatinine plasma levels, and the choice of treatment. Without treatment, 90% of patients die within 3 years, mostly due to complications of liver cirrhosis (13).

Here, we present the first case of rhabdoid meningioma concurrent with a history of Budd-Chiari syndrome and liver cirrhosis. The patient was diagnosed with the BCS at age 38 and the angiography of the inferior vena cava combined with balloon dilatation and anticoagulation as well as hepatoprotective therapy were performed. Unfortunately, when he turned 40 who had magnetic resonance imaging (MRI) that showed a left frontotemporal lobe space-occupying lesion, and postoperative pathological examination confirmed rhabdoid meningioma. He underwent surgery and postoperative adjuvant radiotherapy, but then he developed severe psychiatric symptoms and eventually succumbed to a lung infection two months after treatment.

## Case presentation

**Figure 1:**
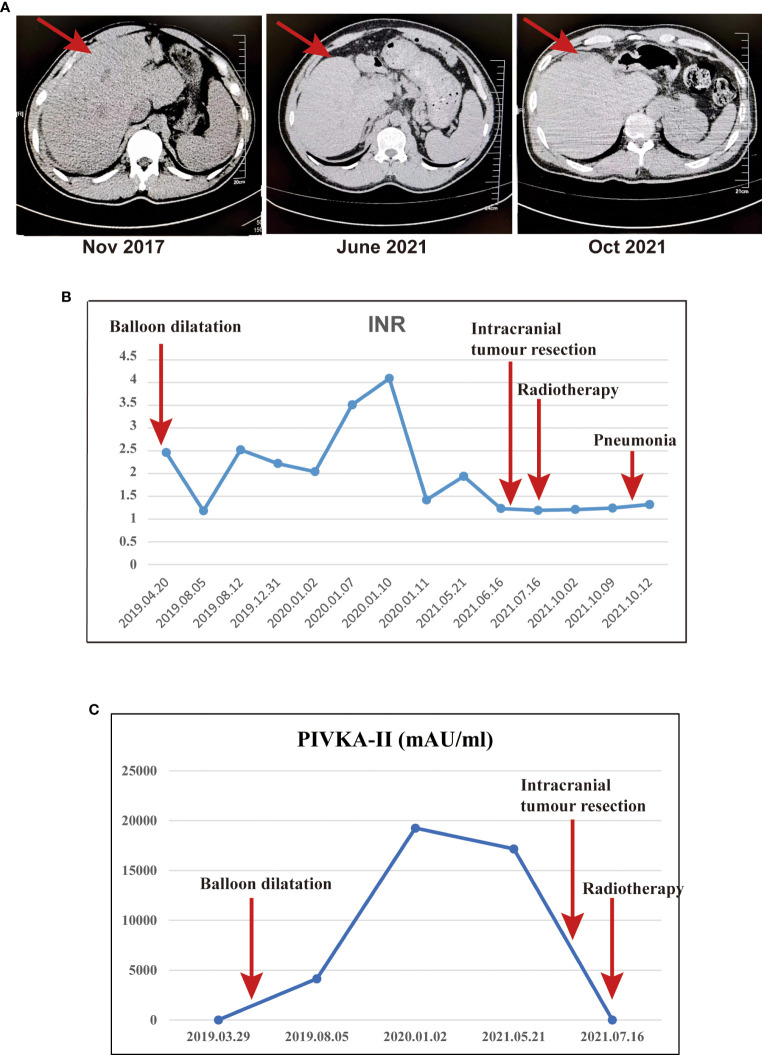
The abdominal CT, INR and PIVKA-II were monitored throughout the course of the disease. **(A)** The abdominal CT indicated early cirrhosis in November 2017, the liver volume decreased significantly in October 2021.; **(B)** The INR throughout the course of the disease. **(C)** The PIVKA-II throughout the course of the disease.

The man was found to have abnormal liver function during physical examination in 2016 at 36 because his liver enzymes levels were as follows: alanine aminotransferase (65 U/L), aspartate aminotransferase (53 U/L) and normal bilirubin, and these were not paid attention to and were not reviewed regularly. In November 2017, the patient came to the First Affiliated Hospital of Kunming Medical University for abdominal CT and indicated early cirrhosis (**Figure 1A**), but no further treatment was given. In early 2019, the patient went to several hospitals in Yunnan Province for treatment due to abdominal pain. The abdominal MRI examination showed cirrhosis with ascites, and the aetiology was not clear after a complete general examination. Then, he was treated with oral polyene phosphatidylcholine, and his abdominal pain still did not improve. In April 2019, the patient went to Beijing YouAn Hospital Affiliated to Capital Medical University for further treatment. The abdominal CT indicated cirrhosis, emboli of the left hepatic vein and Budd-Chiari syndrome. Further angiography of the inferior vena cava showed membranous occlusion of the hepatic segment of the inferior vena cava. Thus, the patient was definitively diagnosed with Budd-Chiari syndrome. Then, angiography of the inferior vena cava combined with balloon dilatation was performed, followed by low-molecular-weight heparin, warfarin and polyene phosphatidylcholine therapy. Low-molecular-weight heparin (LMWH) was stopped, polyene phosphatidylcholine and warfarin continued to be given after the patient’s INR increased to 2.46. Subsequently, liver function indicators and blood clotting mechanisms were regularly monitored to prevent the deterioration of cirrhosis and bleeding. Among them, the core index of anticoagulant therapy INR and a marker of liver cancer PIVKA-II were focus. The INR remained within the safe range throughout the course of the disease, and PIVKA-II continued to rise and decreased to normal in July 2021 (**Figures 1B, C**). In addition, the alanine aminotransferase, aspartate aminotransferase and bilirubin remained within the normal range throughout the course of the disease (**Supplementary Table S1**).

**Figure 2:**
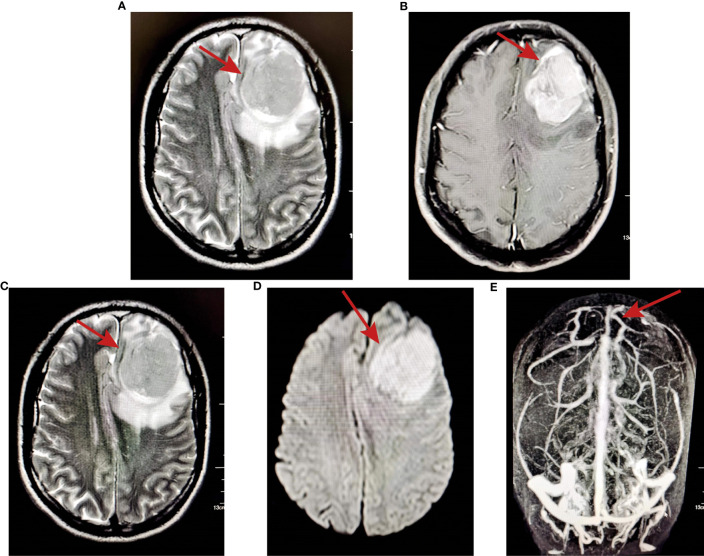
MRI examination showed a space-occupying lesion in the left frontotemporal lobe. **(A)** T2-weighted images; **(B)** T1-weighted images; **(C)** FLAIR showed slightly high signal and the boundary was still clear; **(D)** DWI showed high signal enhancement; **(E)** MRA showed that the compression of the bilateral anterior cerebral artery shifted to the right.

In March 2021, the patient complained of dizziness and headache, accompanied by nausea and vomiting, without numbness or disturbance of movement in the limbs. The patient did not pay attention to these and had not undergone further examination until June 2021. The patient was treated in Yunnan Cancer Hospital for liver cirrhosis; MRI examination found a space-occupying lesion in the left frontotemporal lobe, but further specific diagnosis and treatment were still not performed. Then, the patient developed blurred vision and double vision and went to the First Affiliated Hospital of Kunming Medical University for treatment. Head MRI examination revealed a shallow lobulated round mass (approximately 4.4×4.6×4.9 cm) with slightly long T2 and T1 signals under the left frontal cranial plate. FLAIR showed a slightly high signal, and the boundary was still clear. DWI showed high signal enhancement and obvious enhancement, and the adjacent dura mater was slightly thickened and enhanced. Moreover, the adjacent brain parenchyma was obviously compressed, and a large area of oedema and a right-skewed midline structure were observed. MRA showed that the compression of the bilateral anterior cerebral artery was shifted to the right, the A1 segment of the left anterior cerebral artery was slender, the distal part was well developed, and the M1 segment of the left middle cerebral artery was shifted downwards (**Figure 2**).

**Figure 3:**
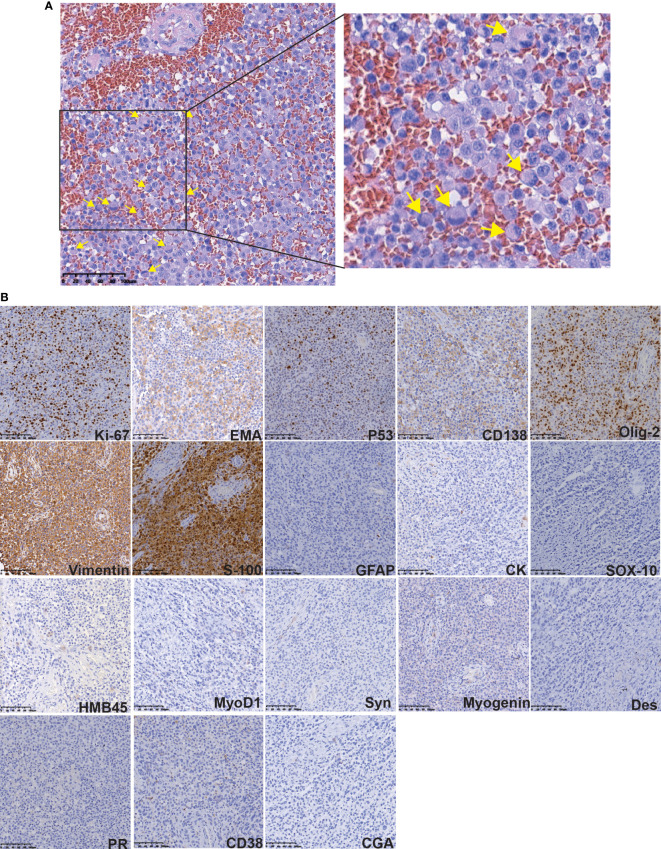
Histopathologic examination after HE and IHC staining of the surgical specimen. **(A)** The HE staining revealed rhabdoid cells features: plump cells with eccentric nuclei, open chromatin, a prominent nucleolus, and prominent eosinophilic paranuclear inclusions (yellow arrow). **(B)** The IHC staining of some protein.

**Figure 4:**
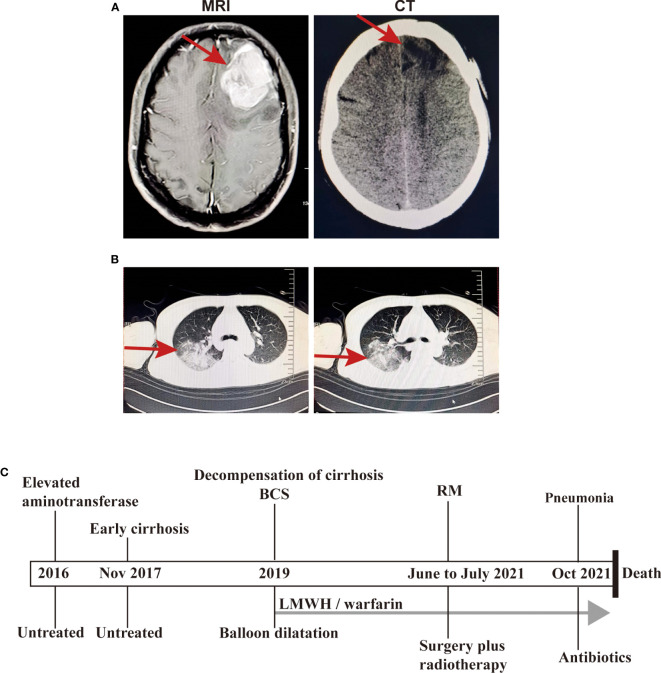
The images at the follow-up phase and the timeline for treatment regimens. **(A)** The head CT showed a low-density image in the left frontal blade after treatment (right) and the MRI T1-weighted image without treatment (left); **(B)** The chest CT scan showed a large flaky blur in posterior segment of lower lobe of right lung and considered pulmonary infection; **(C)** The timeline for treatment regimens.

After imaging and haematology tests, the patient was transferred to the Department of Neurosurgery, warfarin was stopped 1 week preoperatively and perioperative heparin bridging and coagulation function testing were performed because of concern for worsening of the patient’s liver condition during anticoagulation pause. Then, intracranial tumour resection was performed on June 22, 2021. Postoperatively, LMWH was started after the indwelling catheter was removed and warfarin was restarted after the stitches were removed. Throughout the perioperative period, the INR was maintained between 1 and 1.5 (**Figure 1B**). Histopathologic examination after HE staining of the surgical specimen revealed rhabdoid cell features: plump cells with eccentric nuclei, open chromatin, a prominent nucleolus, and prominent eosinophilic paranuclear inclusions (**Figure 3A**). Immunohistochemistry showed that the positive rate of the Ki-67 index was 20-30%, the positive of Vimentin, S-100, EMA, CD138 and Olig-2, and the local positive of wild P53. However, GFAP, CK, SOX-10, HMB45, MyoD1, Syn, CGA, Myogenin, Des, PR and CD38 were negative (**Figure 3B**). Thus, the patient was definitively diagnosed with RM, WHO grade 3. Three weeks postoperation, radiotherapy was performed with a dose of 60 Gy in 30 fractions at the tumour bed (PGTV) and 50 Gy at the subclinical tumour area (PCTV) by using volumetric modulated arc therapy (VMAT) (**Supplementary Figure 1** and **Supplementary Table 2**) plus continuous daily temozolomide (75 mg per square metre of body-surface area per day, 7 days per week from the first day of radiotherapy for 42 days) to increase radiosensitivity. Subsequently, the patient was recruited into the follow-up phase. After 2 months of follow-up, the patient developed unexplained visual and auditory hallucinations, but the head CT showed a low-density image in the left frontal blade which was considered as the postoperative changes, no significant tumour residue or recurrence was observed (**Figure 4A**). On October 10, the patient developed obvious fever, cough, yellow sputum, shortness of breath after being cold. The haematological examination revealed a significant increase in leukocyte (16.05×10^9^/L), neutrophil (14.68×10^9^/L), C-reactive protein (157.8 mg/L), the CT scan showed a large flaky blur in posterior segment of lower lobe of right lung and considered pulmonary infection (**Figure 4B**), and the sputum culture revealed multidrug-resistant Acinetobacter baumannii. Subsequently, cefoperazone sulbactam and meropenem was used for anti-infective therapy for 2 weeks, there was no significant reduction of cough and sputum, and dyspnoea worsened and increased hypoxia. On October 26, the patient gave up treatment and went home. Finally, he died at home on October 30, 2021. The timeline for treatment regimens is summarised in **Figure 4C**.

## Discussion

RM is a rare aggressive meningioma with rhabdomyoblastic features but without rhabdomyoblastic differentiation (4–6). It has a high risk of local recurrence, tumour spread and distant metastasis. This disease can occur at all ages, the onset age of RM is relatively young, and most patients are either young or middle-aged compared with general meningioma patients (14). The incidence rate was not significantly different by sex. The clinical manifestations vary according to the location of RM, mainly manifesting as intracranial hypertension and corresponding nerve compression symptoms (15). The most common sites were the sagittal sinus, posterior cranial fossa, convex cerebri and cerebellopontine angle.

CT and MRI features are not significantly different from those of general meningiomas, which can have hyperenhancement signals and meningeal tail signs. The tumour body and surrounding tissues can exhibit haemorrhage or oedema or even cystic degeneration. EY Kim (14) et al. investigated the CT and MR Findings and clinical features of 15 patients with histologically confirmed RMs and pointed out that the imaging findings of RM often have significant peritumoral oedema, cystic components and bone involvement. In addition, according to the existing reports (16, 17), ^68^Ga-DOTATOC PET/CT imaging can be used as a new assessment tool to improve the preoperative and postoperative evaluation of patients.

Pathological examination with the presence of rhabdoid cells is the gold standard for the diagnosis of RM (1). The differential diagnosis of RM includes malignant melanoma, metastatic carcinomas, atypical teratoma, rhabdoid tumour, and plasmacytoma. Most RMs have characteristic meningioma structures and rhabdoid cells. It is not difficult to make a differential diagnosis by combining the location of the disease and immunophenotypes. In terms of immunophenotypes, epithelial membrane antigen (EMA) and vimentin are positively expressed in RM, but the expression of myogenic markers (mainly actin, desmin, SAM) is mostly negative (5, 6, 18). The histopathological and immunohistochemical findings in this case were consistent with those reported thus far, and in addition, this case showed positive staining for p53 protein. Notably, the positive rate of the Ki-67 index (MIB-1) in the postoperative pathological examination is closely related to tumour proliferation and malignancy (19, 20). The Ki-67 index of the present case was more than 20%, thus, the cancer cells of this patient will proliferate rapidly and are likely to metastasise.

There is still no unified standard for RM treatment. Radical surgery combined with radiotherapy is the main method of treatment and the key to prolonging survival (4). At present, some studies have pointed out that cytotoxic drugs have certain efficacy and clinical value for aggressive meningiomas, but the evidence is limited (21). According to existing reports, oncogenic mutations in the NF2 and AKT1 genes provide alternative approaches for drug therapy (22), and CD105 may be a promising target in stem cell therapy (15). Early conventional radiotherapy is recommended for malignant meningiomas regardless of whether surgical resection is complete. Tumour recurrence is mainly related to the extent of surgical resection. It has been reported that sunitinib maleate (18) and gamma knife stereotactic radiosurgery (23) can be used as adjuvant treatments to prevent recurrence. Future drug treatment approaches are mainly based on the identification of potential therapeutic targets, such as *AKT1*, *SMO*, and *PD-1/PD-L1 (* 24).

Budd-Chiari syndrome (BCS) is defined as obstruction of the hepatic venous outflow tract without right heart failure or constrictive pericarditis (7). The obstruction causing BCS may be located in the small or large hepatic veins or in the suprahepatic portion of the inferior vena cava (IVC), but does not include sinusoidal obstruction syndrome/hepatic veno-occlusive disease (11). It was first described in 1845 by a British physician, William Budd, in his book Diseases of the Liver (25).

The pathogenesis of Budd-Chiari syndrome in Western countries is significantly different from that in China (26, 27). In Western countries, it is mainly caused by lesions of the hepatic vein and is closely related to the hypercoagulable state of the body, and myeloproliferative disease is the most common cause (8). However, in China, lesions of the inferior vena cava membrane are more common, and the venous diaphragm shape is the characteristic pathological change (26).

The clinical symptoms of BCS lack specificity because of the diversity of clinical manifestations and onset forms, which range from asymptomatic to fulminant liver failure (7). Thus, it is easily confused with cirrhosis caused by other causes, portal hypertension, peritonitis and other diseases. Abdominal pain, distention, and hepatomegaly are considered the typical triad of BCS, but abdominal pain is a less common finding, and splenomegaly is more common (28). The clinical presentation depends on the degree and speed of hepatic venous outflow obstruction and the presence or absence of venous collateral circulation to decompress the hepatic sinusoids (27). Although BCS is rare, it should be suspected in a variety of acute and chronic liver diseases (11, 27, 28).

BCS is most easily diagnosed by identifying obstruction of the hepatic venous system on imaging (8). The main imaging examinations included ultrasound, CT, MRI, inferior vena cava and hepatic vein angiography. Invasive hepatic venography and venography of the caval are the “gold standard” for the diagnosis of BCS, but they are not suitable for routine diagnosis of suspected clinical cases (28). Colour Doppler ultrasound is the preferred diagnostic method for BCS screening and can accurately display the location, range and degree of obstructive vessels in patients; moreover, it can display haemodynamic information without contrast agents with high safety and repeatability. CT and MRI can provide more comprehensive intrahepatic and extrahepatic information to clarify the classification of BCS and evaluate the nature of thrombosis and collateral circulation, which is helpful for early diagnosis and appropriate treatment.

The purpose of BCS treatment is to relieve hepatic venous outflow obstruction, relieve hepatic venous pressure, improve liver function and prevent further thrombosis (27). Current treatment options include drug therapy, such as anticoagulants and diuretics, and invasive therapies (29), which mainly include thrombolysis, percutaneous transluminal angioplasty, transjugular intrahepatic portosystemic shunt, surgical portosystemic shunt and orthotopic liver transplantation. When BCS develops to an advanced stage, liver transplantation is the only option. The goal of anticoagulation is to avoid the expansion of the thrombus, to maintain the latency of spontaneous recanalisation of the hepatic venous outflow tract as much as possible, and to avoid thrombus formation at other sites (28). Therefore, regardless of whether potential prethrombotic disorders have been identified, it is recommended that all patients with BCS start anticoagulant therapy early (30). For patients with Budd-Chiari syndrome and long-term oral anticoagulants, we should closely monitor their coagulation function in the subsequent treatment, including surgery, radiotherapy and chemotherapy.

In conclusion, Budd-Chiari syndrome and rhabdoid meningioma are both extremely rare, and there is no report that the two diseases occurred in the same patient. Herein, we showed an unusual case of rhabdoid meningioma with a history of Budd-Chiari syndrome. To the best of our knowledge, the patient described in this report presents the first case of a patient who suffered from rhabdoid meningioma and Budd-Chiari syndrome. The occurrence of these two disorders in the same patient is puzzling, and there is no definitive evidence that the two diseases are related or to rule out chance in this case.

## Data availability statement

The original contributions presented in the study are included in the article/**Supplementary Material**. Further inquiries can be directed to the corresponding authors.

## Ethics statement

Written informed consent was obtained from the patient ‘s family for the publication of the case report and all accompanying images.

## Author contributions

YiZ and YoZ were involved in the identification and selection of patient cases and drafted the manuscript. YY was involved in the HE and IHC detection as well as pathological diagnosis. RL and JZ reviewed and edited the manuscript. WJ, JZ and QF were involved in the patient’s clinical management. YiZ and QF were involved in the identification, selection and management of patient cases, and reviewed and edited the manuscript. RL and QF confirm the authenticity of all the raw data. All authors contributed to the article and read and approved the final version.

## Supplementary material

Supplementary Figure 1 The image with Volumetric Modulated Arc Therapy (VMAT) dose prescription by linear accelerator. **(A)** Transverse section showed the PGTV (Planning Gross Tumour bed Volume) (red line) and PCTV (Planning Clinical Tumour Volume) (yellow line) regions; **(B)** Median sagittal section showed the PGTV (red line) and PCTV (yellow line) regions; **(C)** Dose distribution across brain in temporoparietal region using 6 MeV X-rays; **(D)** Assessment of Feasibility dose-volume histograms (DVH) for head treatment planning.
