# Supplementary figures and images for: Rhabdoid meningioma with a history of Budd-Chiari syndrome: a case report and review of the literature
Source: Front Oncol. 2023 Jul 11;13:1209244. doi: 10.3389/fonc.2023.1209244 (PMC10370419; doi:10.3389/fonc.2023.1209244)

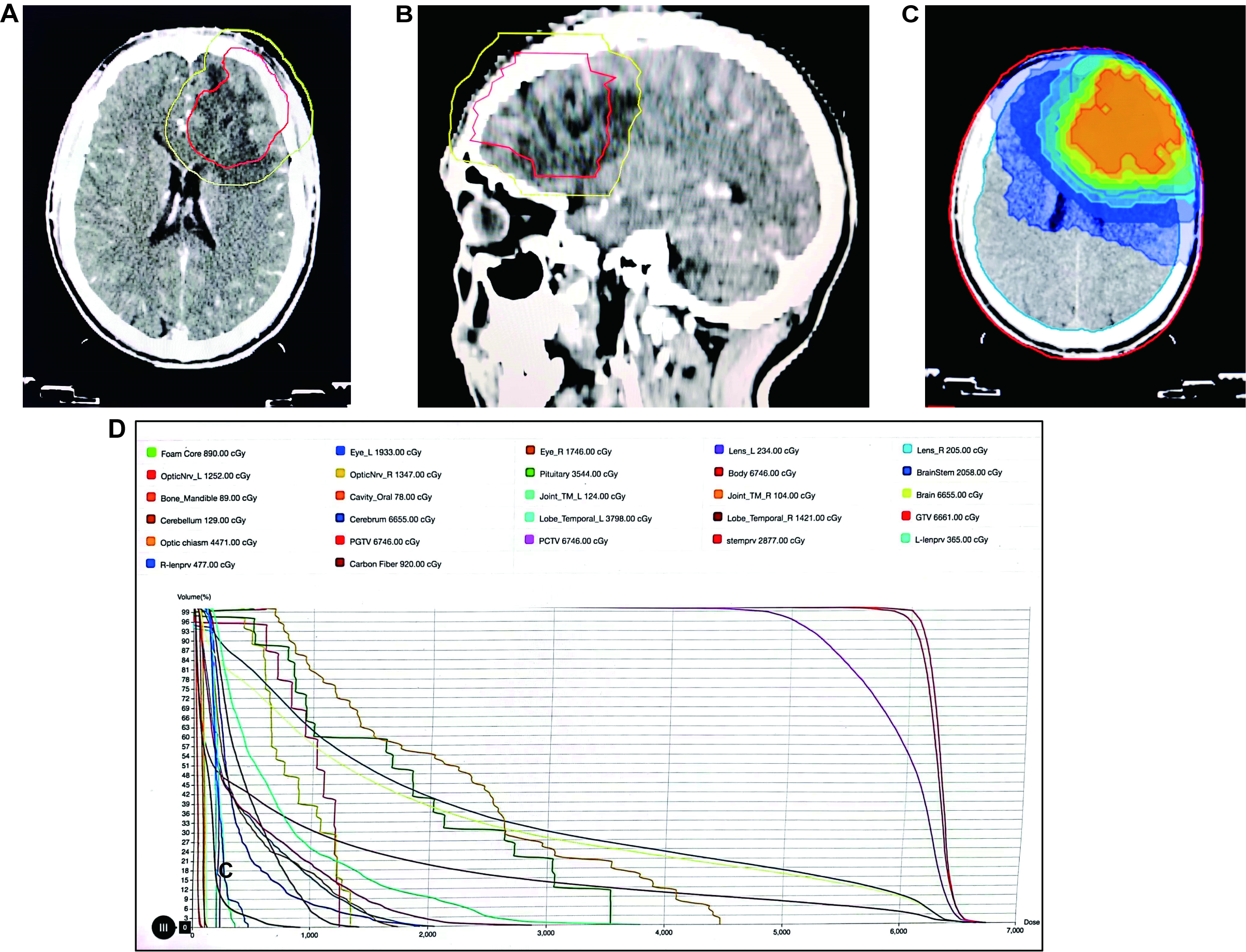

Supplement: Supplementary Figure 1 — The image with Volumetric Modulated Arc Therapy (VMAT) dose prescription by linear accelerator. (A) Transverse section showed the PGTV (Planning Gross Tumour bed Volume) (red line) and PCTV (Planning Clinical Tumour Volume) (yellow line) regions; (B) Median sagittal section showed the PGTV (red line) and PCTV (yellow line) regions; (C) Dose distribution across brain in temporoparietal region using 6 MeV X-rays; (D) Assessment of Feasibility dose-volume histograms (DVH) for head treatment planning. [file Image_1.tif]
