# Supplementary material for: Rhabdoid meningioma with a history of Budd-Chiari syndrome: a case report and review of the literature
Source: Front Oncol. 2023 Jul 11;13:1209244. doi: 10.3389/fonc.2023.1209244 (PMC10370419; doi:10.3389/fonc.2023.1209244)
Supplement: Supplementary file 3 [file Table_2.docx]

Table 2 The doses of the target area and organ-at-risk dose (OAR) by using volumetric modulated arc therapy (VMAT).

| Target area  and OAR | Dose of Dv95%  (Gy) | Maximum dose  (Gy) | Minimum dose  (Gy) | Mean dose  (Gy) |
| --- | --- | --- | --- | --- |
| PGTV | 60.03 | 67.51 | 53.96 | 62.51 |
| PCTV | 50.61 | 67.51 | 41.63 | 59.23 |
| Brain Stem |  | 22.08 | 0.41 | 4.61 |
| Pituitary |  | 39 | 5.02 | 19.02 |
| Left Lens |  | 2.74 | 1.6 | 2.02 |
| Right Lens |  | 2.1 | 1.58 | 1.82 |
| Left Optic nerve |  | 12.7 | 6.04 | 10.30 |
| Right Optic nerve |  | 13.86 | 4.23 | 8.90 |
| Left Eye |  | 19.47 | 1.22 | 4.10 |
| Right Eye |  | 17.72 | 1.15 | 4.85 |
| Optic Chiasm |  | 45.07 | 6.79 | 22.37 |
